# Supplementary material for: Distribution of naturally -occurring NS5B resistance-associated substitutions in Egyptian patients with chronic Hepatitis C
Source: PLoS One. 2021 Apr 15;16(4):e0249770. doi: 10.1371/journal.pone.0249770 (PMC8049381; doi:10.1371/journal.pone.0249770)
Supplement: Attachment — Submitted filename: Reponse_Plos.docx [file pone.0249770.s001.docx]

In this manuscript authors analyzed the RASs occurrence in 17 treatment-naïve and 1 non-responder samples out of 35 total number of patients. Samples were collected from patients who failed to achieve an early viral response with combined sofosbuvir (400mg/day), daclatasvir (60mg/day) for 6 months therapy. Authors reported several mutations as possible RASs linked to SOF.

Although these mutations may be of a great value to understand DAA resistance, however, the study lack important aspects that need to be addressed to be publishable. Examples for these shortcomings are the small number of the analyzed samples especially the non-responders, lack of comparison with other cohort of patients who achieved an early viral response, and the use of multiple DAAs but analyzing one target gene. Although daclatasvir targets NS5A, nothing was done on this gene as patients may be resistant to daclatasvir and this resistance was related to NS5A and not NS5B. The manuscript has many places that need rephrasing or improvement to read better. In addition, the references need to be updated as the authors did not include several important and recent papers about the topic.

More detailed comments will be mentioned below.

**Title**

- For “Distribution of naturally occurring NS5B resistant associated substitutions among treatment- naïve HCV Egyptian patients and non-responders”

The title is not accurate as the authors should not consider the non-responders data as it came out from only one patient.

**Abstract**

- Authors mentioned “Results: NS5B amino acid sequence analysis showed the presence of several novel NS5B mutations (more than 40 substitution mutations) that have not been previously reported to be associated with a resistant phenotype.”

Without comparing with another cohort with EVR, these mutations may be not related to resistance.

- “The major S282T mutation inducing a high level of resistance to sofosbuvir as well as other reported mutations (L320F/C) and (C316Y/N) was not detected.”

Change to were not detected.

**Introduction**

- “Hepatitis C virus (HCV) is a serious public health concern which affects 3% of the world’s population (130 million people) and one million new cases reported annually **(****1****).”**

I do not think this is the rate now after applying the new treatment. Can you update this information from more recent references?

- “NS5B acts as an RNA-dependent RNA polymerase (RdRp) which synthesizes RNA using a RNA template (10, 11).”

Should be an RNA template.

**Results**

- “For non-responders, it was found that 5/8 patients showed normal levels of all tested variables. On the other, one patient showed abnormal results for all tested variables .”

What about the remaining 2/8, are they normal or not? It is advisable to include the value of what is considered normal in the title of each column in the table (1).

- “In this study, the success rate of amplification for NS5B fragments was 17/27 of the collected treatment naïve ………………………longer turnaround time (22-24).(23).”

Remove (23).

- Change sentence to “Most treatment- naïve patients in this study were genotyped as HCV genotype-4a 13/17 (76.5%), Subtype 3a and 4l were each only identified in one sample (5.9%), …….”
- In the paragraph started with “Ribavirin-associated resistance mutations at positions D244N, Q309R, and A333E of NS5B were analyzed.” I am not sure why the authors compare their mutations with non-DAAs drugs and ignore comparing them with other DAAs drugs. Either they include the most related drugs or remove this paragraph altogether.
- Table 1, It is not clear why the authors did not arrange ID numbers in order. This has to be arranged correctly or explained by the authors.
- Also, in Table 1, non-responders showed low viral load in comparison to naïve patients. This has to be addressed and reasonable explanation has to be made.
- In Table 2. Change no. of strains to no. of cases.
- In Table 4, it is not clear why the authors arrange ID numbers or the mutations this way. Neither IDs, number of mutations, or location of the mutations are in order. I recommend to order the table according to the no. of substitutions as well as the locations of the mutations. Each column should include one location and if at that position different mutations exist, it should be market with asterisk. Example, first column should be only for position 231 with asterisk for A231G* and moving 244 to different column in order.
- In this paragraph “Regarding samples with multiple mutations, it was observed that sample with ID 10 was found to have 13 substitution mutation sites whose patient of 58 years old showing normal clinical characteristics. Patient’s sample with ID 2 showed 12 substitution mutation sites showing only abnormal level of ALT. Patient’s sample with ID 15 showed 11 substitution mutations sites, was found to have only abnormal level of ALT. In addition, variable clinical characteristics were observed in patients’ samples IDs 8, 12, 16, 17 representing 10 mutations, which indicates that no relations among the number of substitution and the clinical characteristics of the patients.”

All the referred numbers of substitutions to ID numbers are wrong and need corrections according to Table 4.

**Discussion**

- The Second paragraph discuss the abundance of 4a subtype in agreement with other works. This part is irrelevant to this study as this is a well-documented fact and did not add much to this study.
- Surveillance of populations to identify the baseline prevalence of HCV DRMs , such as S282T, is….

Spell out DRMs.

- “In our study relevant natural aa polymorphisms were found in genotypes 4a, 4l, 3a, and 1g were found,”

Remove last “were found” and replace “,” with “.”

- For this paragraph “**Kouyoumjian, Chemaitelly (30)** showed that genotype 4a represent 12.06% of Egyptian HCV while 41 and 4o genotypes represents 0.24% and 0.91%, respectively. Abdel-Hamid, El-Daly (22) revealed that 4o genotype was strongly associated with Hepatocellular carcinoma (HCC) in chronic HCV patients while in this study the only amplified sample of the non-responder patient was 4o genotype.”

What is the importance of this paragraph to the current study? And how come 4a represent only 12% if it is the most dominant subtype. According to this percentage there is other subtypes that is more abundant than 4a. I recommend to explain more about the reason of adding these information or remove it altogether as they are irrelevant to the study.

- For “Finally, the sustained virological response in most patients including those carrying DAA resistance associated substitutions (RASs) may be due to virus clearance.”

Rephrase, as it seems that RASs are carried by the patients and not in the virus. Also how the virus will be cleared out while it is resistant to DAAs?

- For “The role of theses RASs should be evaluated in the patients receiving DAA interferon free regimens or those who will receive treatment soon to know the role of these RASs in modulating the viral resistance level or the susceptibility to currently used drug regimens”

I thought that the current study already did that and evaluated patients receiving DAA interferon-free regimens as indicated in the results.

**Citations**

Authors missed several references that may have helped their discussion and conclusion, especially that few publications addressed G4. Suggested references:

Fourati S, Rodriguez C, Hézode C, et al. Frequent antiviral treatment failures in patients infected with hepatitis C virus genotype 4, subtype 4r. Hepatology. 2019;69(2):513-523.

Ji H, Kozak RA, Biondi MJ, et al. Next generation sequencing of the hepatitis C virus NS5B gene reveals potential novel S282 drug resistance mutations. Virology. 2015;477:1-9.

N Fadl, TZ Salem. Hepatitis C genotype 4: A report on resistance‐associated substitutions in NS3, NS5A, and NS5B genes. 2020, Reviews in Medical Virology 30 (4), e2120.

Raj VS, Hundie GB, Schürch AC, et al. Identification of HCV resistant variants against direct acting antivirals in plasma and liver of treatment Naïve patients. Sci Rep. 2017;7(1):4688.

Gane et al., 2017. The emergence of NS5B resistance associated substitution S282T after sofosbuvir‐based treatment.

Catalli et al., 2019. Novel NS5B Resistance‐Associated Substitution Emerges Under Failing Sofosbuvir/Ledipasvir Therapy.

General Minor:

- Change all treatment naïve to “treatment-naïve”
